# Supplementary material for: Micronuclei in Circulating Tumor Associated Macrophages Predicts Progression in Advanced Colorectal Cancer
Source: Biomedicines. 2022 Nov 11;10(11):2898. doi: 10.3390/biomedicines10112898 (PMC9687174; doi:10.3390/biomedicines10112898)
Supplement: Supplementary file 1 [file biomedicines-10-02898-s001.zip › biomedicines-1947967-supplementary.pdf]

	Univ. HR (PFS)	Univ. P-Value (PFS)	Multi. P-Value (PFS)	Univ. HR (OS)	Univ. P-Value (OS)	Multi. P-Value (OS)
**Age**	1.484	0.7671	-	1.775	0.7322	-
**Gender**	2.372	0.3201	-	1.559	0.8645	-
**Metastatic vs Non-Metastatic**	5.892	0.1272	-	12.556	**0.0056**	0.9975
**MN Presence**	17.148	**0.0014**	-	70.336	**0.0036**	0.9981
**Histology**	3.404	0.110	-	2.874	0.3459	-

Table S1. Univariate and Multivariate analysis for clinically available variables. Univariate analysis was conducted for each available clinical variable. For PFS and OS, MN presence was found to be significant (bolded). For OS, metastatic presence (stage IV) and non-metastatic presence was compares and found to be significant.

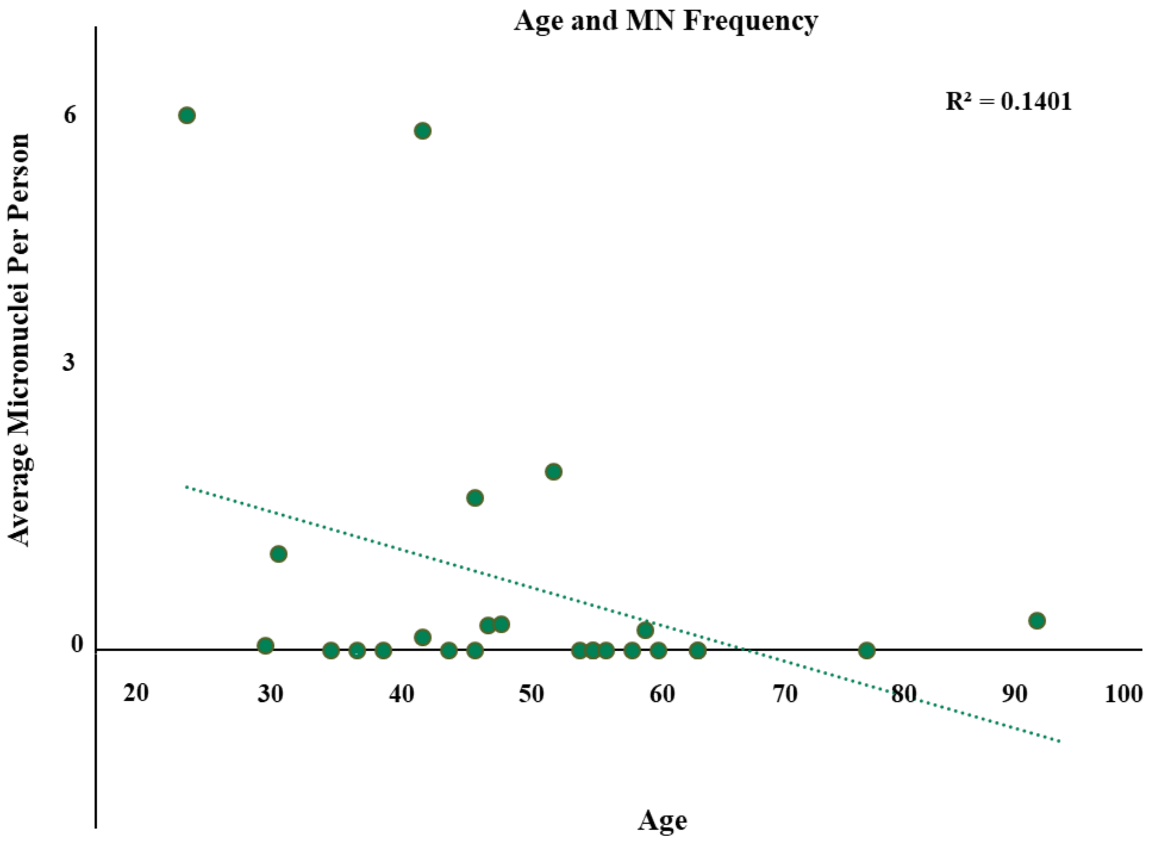

Figure S1. Age vs MN Frequency. Linear regression plot of the relationship of patient age versus MN Frequency each patient at baseline sampling.
